# High rates of rock organic carbon oxidation sustained as Andean sediment transits the Amazon foreland-floodplain

**DOI:** 10.1073/pnas.2306343120

**Published:** 2023-09-19

**Authors:** Mathieu Dellinger, Robert G. Hilton, J. Jotautas Baronas, Mark A. Torres, Emily I. Burt, Kasey E. Clark, Valier Galy, Adan Julian Ccahuana Quispe, A. Joshua West

**Affiliations:** ^a^Department of Geography, Durham University, Durham DH1 3LE, United Kingdom; ^b^Environnement, Dynamique et Territoires de Montagne, CNRS-University Savoie Mont Blanc, Le Bourget du Lac 73373, France; ^c^Department of Earth Sciences, University of Oxford, Oxford OX1 3AN, United Kingdom; ^d^Department of Earth Sciences, Durham University, Durham DH1 3LE, United Kingdom; ^e^Department of Earth, Environmental, and Planetary Sciences, Rice University, Houston, TX 77005; ^f^Department of Earth Sciences, University of Southern California, Los Angeles, CA 90089; ^g^Department of Geography & Planning, University of Liverpool, Liverpool L69 7ZT, United Kingdom; ^h^Department of Marine Chemistry and Geochemistry, Woods Hole Oceanographic Institution, Woods Hole, MA 02543; ^i^Escuela Profesional de Biologia, Facultad de Ciencas, Universidad Nacional de San Antonio Abad del Cusco, Cusco 08000, Peru

**Keywords:** carbon cycle, weathering, organic carbon, Amazon, rivers

## Abstract

The oxidation of organic carbon contained within sedimentary rocks (“petrogenic” carbon, or hereafter OC_petro_) emits nearly as much CO_2_ as is released by volcanism, thereby playing a key role in the long-term global C budget. High erosion rates in mountains have been shown to increase OC_petro_ oxidation. However, these settings also export unweathered material that may continue to react in downstream floodplains. The relative importance of OC_petro_ oxidation in mountains versus floodplains remains difficult to assess as disparate methods have been used in the different environments. Here, we investigate the sources and fluxes of rhenium (Re) in the Rio Madre de Dios to quantify OC_petro_ oxidation from the Andes to the Amazon floodplain using a common approach. Dissolved rhenium concentrations (n = 131) range from 0.01 to 63 pmol L^−1^ and vary depending on lithology and geomorphic setting. We find that >75% of the dissolved Re derives from OC_petro_ oxidation and that this proportion increases downstream. We estimate that in the Andes, OC_petro_ oxidation releases 11.2^+4.5^/_−2.8_ tC km^−2^ y^−1^ of CO_2_, which corresponds to ~41% of the total OC_petro_ denudation (sum of oxidized and solid OC_petro_). A Re mass balance across the Rio Madre de Dios shows that 46% of OC_petro_ oxidation takes place in the Andes, 14% in the foreland-lowlands, and 40% in the Andean-fed floodplains. This doubling of OC_petro_ oxidation flux downstream of the Andes demonstrates that, when present, floodplains can greatly increase OC_petro_ oxidation and CO_2_ release.

## Significance

Erosion and weathering play key roles in Earth’s carbon cycle, controlling climate over millions of years by transferring CO_2_ to and from the atmosphere. Weathering of sedimentary rocks in mountains can release CO_2_ when ancient organic carbon in rocks is chemically broken down. However, mountain erosion also moves rock organic carbon that has not been broken down and is transported by rivers to lowland floodplains to an unknown fate. Here, we use rhenium in rivers to quantify rock organic carbon weathering in the Andes and adjacent floodplain. Erosion in the Andes leads to high rates of CO_2_ release. However, CO_2_ release doubles when including the floodplains. The presence or absence of floodplains next to mountains governs CO_2_ release by weathering.

Substantial climatic changes have occurred throughout geologic time, with the evolution of the carbon cycle and the planet’s habitability modulated by the interplay between tectonic, climatic, erosional and biological processes. Over long timescales (>10^5^ y), the abundance of CO_2_ in the atmosphere is determined by the balance of the major carbon sources and sinks (1). It has been widely debated how the Earth system responds to transient imbalances in the long-term carbon (C) cycle, with much focus of prior research on the CO_2_ sink associated with the chemical weathering of silicate minerals (1, 2). However, recent studies have highlighted the potential importance of changes in CO_2_ fluxes linked to oxidative weathering (OW) reactions of sedimentary rocks on the continents (3–5). Although receiving less attention to date than silicate weathering, OW of reduced phases in sedimentary rocks is central to both the C and O cycles (6, 7). Carbon dioxide release and O_2_ consumption can result from both i) oxidation of fossil (i.e., “rock-derived” or “petrogenic”) organic matter (OC_petro_) and ii) the OW of sulfides that produces sulfuric acid, which can be neutralized by carbonate minerals, or by the carbonate buffer of continental waters leading to net CO_2_ release to the atmosphere (8, 9).

The export of particulate OC_petro_ by rivers in their sediment load has been documented in many places (10–16), but only a few field-based studies have estimated the rate of OW of OC_petro_ (3). The highest fluxes of OW of OC_petro_ (reported as a yield per catchment area, of 5 to 30 tC km^−2^ y^−1^) have been measured in small high standing islands composed of sedimentary rocks such as in Taiwan (17) and New Zealand (4), as well as in catchments from major mountain ranges (0.5 to 5 tC km^−2^ y^−1^) like the Garhwal Himalaya (18) and Swiss Alps (19), and in the Mackenzie River basin (20). These high OW fluxes reflect the role of physical erosion rate in mountains that can continuously supply OC_petro_ to oxygenated surface waters and the atmosphere (17, 21). A consequence is that, in combination with carbonate weathering by sulfuric acid, CO_2_ release by OC_petro_ oxidation can be larger than CO_2_ consumption by silicate weathering in many erosive settings (3), challenging the idea that chemical weathering in mountain belts is a long-term carbon sink (22–24).

While physical erosion can enhance the supply of reduced phases to OW reactions and increase the weathering flux (8, 17, 25), the overall intensity of weathering can also decline (26), meaning that erosive catchments can also export very large amounts of “unweathered” particulate OC_petro_ (7). These particulate fluxes can be very high, for instance reaching 250 tC km^−2^ y^−1^ (13, 26). The subsequent fate of this material is critical to governing how physical erosion and weathering impact the carbon cycle. It has been proposed (10, 14) that this OC_petro_ can be largely oxidized in floodplains when present (e.g., in the Amazon and Ganges floodplains), because of the long residence time of sediments and the warm and oxidative conditions that prevail in the floodplains relative to the upstream mountainous area. Hence, mountain catchments which are underlain by sedimentary rocks with river floodplains could have greater net CO_2_ release from OW than mountain catchments without floodplains. Over 10^5^ to 10^6^ y timescales, the configuration of continental drainages can shift dramatically (27) and young, high standing mountain ranges potentially operate differently in terms of OW and CO_2_ release relative to mountain ranges with floodplain drapes.

Despite the recognition of these geomorphological controls on OW (3), the relative contributions of mountain versus floodplain weathering to OC_petro_ oxidation and CO_2_ release remains unknown. This knowledge gap arises in part because different methods have been used to assess OW of OC_petro_ in mountain catchments (4, 17) versus floodplains (10, 14). In mountain catchments, dissolved rhenium (Re) fluxes have been used for quantifying OC_petro_ oxidation fluxes (17), whereas studies on floodplains have used sediment fluxes and radiocarbon content of river sediments (10). Both methods have benefits and drawbacks. Dissolved Re fluxes are easier to measure because water discharge is usually known with more precision than sediment flux. On the other hand, there are large uncertainties on the source of dissolved Re and on the estimation of the Re/OC_petro_ ratio of the bedrock (20). Because water and sediments integrate over different spatial and temporal scales, their direct comparison can also be problematic. Overall, the influence of floodplains on the global OC_petro_ budget remains an open question (3, 28).

To resolve these issues, here we investigate the source and fluxes of dissolved and particulate rhenium (Re) in the Madre de Dios watershed, part of the larger Amazon Basin. We focus on an elevation transect ranging from the high Andes to the low-elevation floodplains and use Re to quantify the flux of CO_2_ released by OC_petro_ oxidation. These catchments have been well-characterized by previous studies in terms of erosion rates and their major element dissolved load (11, 23, 29), and benefit from time-series samples over a range of river discharges. Most importantly, this setting provides an opportunity to determine the rate of OC_petro_ oxidation in each of the different geomorphological settings (mountain, foreland, floodplain) within a single river basin.

## Study Site

The Madre de Dios watershed (124,231 km^2^ at the confluence with the Rio Beni in Bolivia) is one of the main headwaters of the Amazon Basin (30), the largest river basin in the world. The precipitation in the Madre de Dios region is between <1,000 and >6,000 mm y^−1^ and the mean annual temperature varies with elevation from 4 to 25 °C (30–32). The Rio Madre de Dios drains: i) the eastern flank of the Andes in Peru with elevations from 1,300 m to 6,300 m, ii) the mountain front with elevations from 500 m to 1,300 m, iii) the foreland corresponding to elevations of less than 500 m, in which river basins are not Andean-fed but have undergone some recent uplift associated with the Fitzcarrald Arch, and iv) the Andean-fed floodplains, which are the low-relief environments adjacent to active river channels from rivers originating from the Andes and where sediments are deposited and exchanged with the main channel. The bedrock in the headwaters is primarily composed of Paleozoic and Mesozoic sedimentary and metasedimentary rocks [with (OC_petro_) ~ 0.4 to 0.6%] and some minor granitic intrusions in the Andes (23). Observations from the neighboring Beni catchment with similar lithologies to the Madre de Dios show that this OC_petro_ is a mixture of disordered and graphitic carbon (10).

**Fig. 1.**
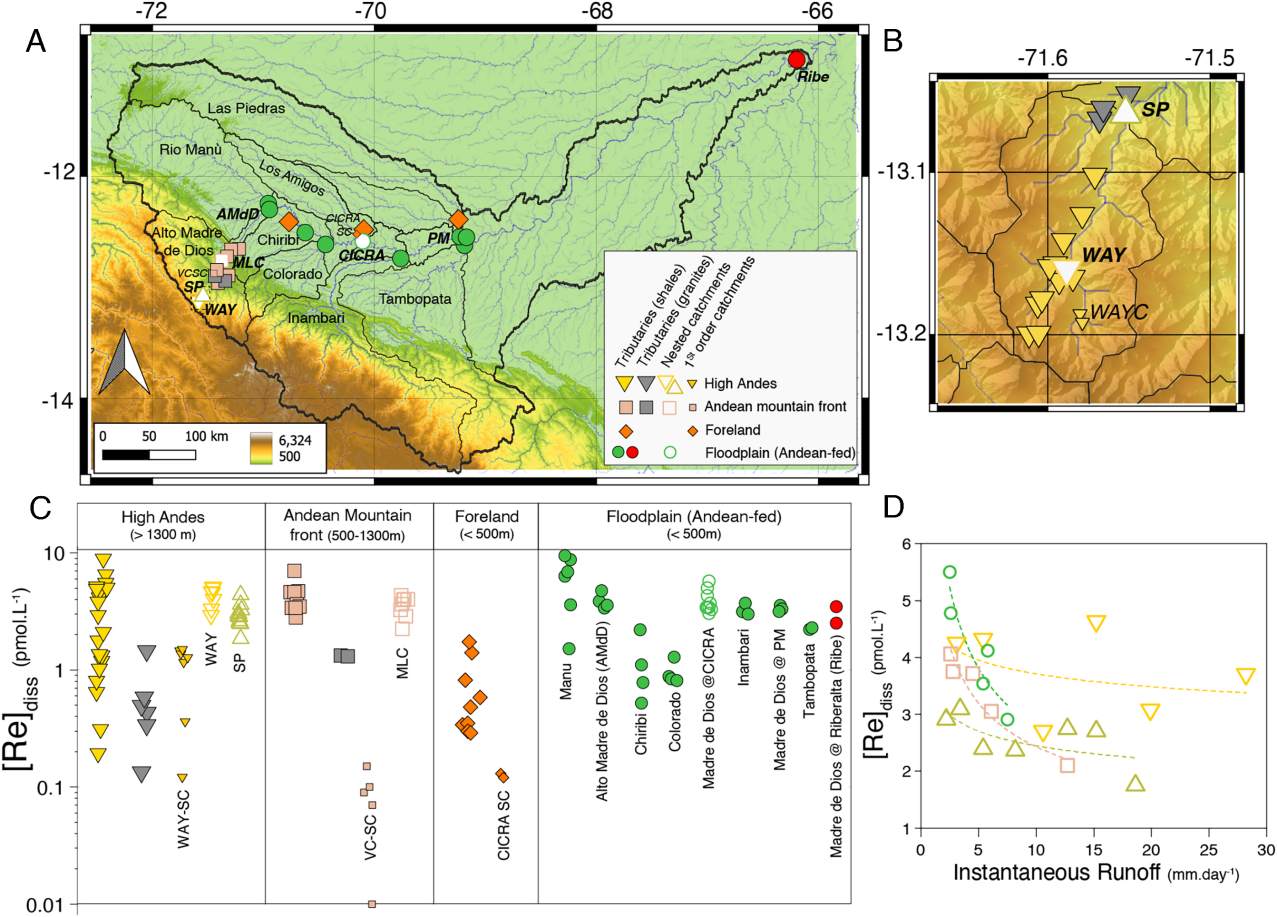
(*A*) Map of the Madre de Dios River basin with the location of the samples from this study. (*B*) *Inset* showing the location of the samples in the High Andes. (*C*) Re concentration (in pmol L^−1^) in the various geomorphic settings for all river samples from this study. The [Re]_diss_ from the two hot spring samples plot above the range shown on the figure (see data in Dataset S1). (*D*) [Re]_diss_ versus instantaneous runoff W (water discharge normalized by catchment area, mm day^−1^) at the time of sampling for the main nested catchments of this study (WAY, SP, MLC, and CICRA).

To characterize the spatial variability of dissolved Re concentrations ([Re]_diss_) across the Madre de Dios River basin (Fig. 1*A*), we studied a large number of small tributaries draining each geomorphological setting that have been subject to prior work on water, sediment, and carbonate and silicate weathering fluxes (11, 23, 29, 33–35). To characterize the dissolved Re sources, we sampled stream, spring, and lysimeter waters from small first-order catchments in each geomorphological setting (referred as Wayqecha Small Catchment “WAY-SC,” Villa Carmen Small Catchment “VC-SC” and CICRA Small Catchment “CICRA-SC”; ref. 36). To examine the downstream evolution of OC_petro_ weathering processes across these geomorphic settings, we studied in detail four main nested subcatchments that define a geomorphic gradient from the Andes to the foreland-floodplain: the Rio Kosñipata at Wayqecha in the Andes (referred as “WAY,” altitude 2,250 m); the Rio Kosñipata at San Pedro in the Andes (“SP,” altitude 1,360 m); the Alto Madre de Dios at Manu Learning Center (“MLC,” altitude 479 m) at the transition between the Andes and the foreland; and the Madre de Dios at CICRA-Los Amigos research station (“CICRA,” altitude 217 m). Time series [Re]_diss_ and discharge data from the years 2010 to 2011 are used to characterize changes in Re concentration during the hydrological cycle. The measured annual runoff for the year 2010 to 2011 was 3,065 mm y^−1^ at the WAY site (Upper Andes) and 2,796 mm y^−1^ at SP (33). The suspended sediment flux (a proxy for erosion rate) at San Pedro (SP) measured during the same period was 3,500 t km^−2^ y^−1^ (11), similar to the estimated suspended sediment load from the Andean area of the entire Madre de Dios watershed (3,208 t km^−2^ y^−1^) (37). Finally, to integrate weathering processes over large areas and establish a Re mass-budget, we studied several large tributaries with Andean-fed floodplains that drain all geomorphic settings (Rios Manú, Colorado, Chiribi, Inambari, and Tambopata).

## Results

Rhenium concentrations were measured on water samples collected from several field campaigns, including i) from the Madre de Dios mainstem and major tributaries sampled under different flow conditions in 2012 and 2013 (23, 34, 38), and again at high and low flow in March and May 2019 (35), ii) from time-series samples collected at four nested catchment sites (areas 50 to 27,830 km^2^) in 2010 and 2011 during different hydrological conditions (29, 33), and iii) from another time series of samples collected from smaller first-order catchments (<1 km^2^ area) across the elevation gradient (36). The time-series dataset is used to characterize the relationship between Re concentration and instantaneous runoff.

### Spatial Variability of Dissolved Re Concentrations.

Rhenium concentrations in dissolved samples (n = 133) range from 0.01 to 63 pmol L^−1^, with spatial variability depending on the sample type, bedrock lithology, and geomorphic setting (Fig. 1*C* and Dataset S1). Rivers have [Re]_diss_ ranging from 0.1 to 9.5 pmol L^−1^. The rainwater sample collected in the Andes has [Re]_diss_ below detection limit ([Re]_diss_ < 0.05 pmol L^−1^) while [Re]_diss_ in the throughfall sample from the same location is 0.36 pmol L^−1^. Lysimeter samples from first-order small catchments in the Andes and in the foreland also have very low [Re]_diss_, ranging from 0.09 to 0.12 pmol L^−1^ (n = 4). In contrast, the two hot spring samples from Aguas Caliente have very high [Re]_diss_ (39 pmol L^−1^ and 63 pmol L^−1^). However, the [Re]_diss_ of the Alto Madre de Dios downstream of the hot springs is unchanged compared to [Re]_diss_ upstream of the hot springs, suggesting that their contribution to the Re budget is negligible.

In terms of lithology, small rivers draining mostly granitic rocks in the High Andes have lower Re concentrations (0.56 ± 0.44 pmol L^−1^, 1σ, n = 6) than rivers draining sedimentary rocks (2.90 ± 2.06 pmol L^−1^, 1σ, n = 25). Rivers draining the mountain front composed of mostly granites also have lower [Re]_diss_ (1.32 pmol L^−1^, n = 2) than those draining sedimentary rocks (3.88 ± 1.12 pmol L^−1^, 1σ, n = 10).

We observe a notable geomorphic control on [Re]_diss_ for the tributaries and first-order catchments draining specific settings, but not for the nested catchments (Fig. 1*C*). Tributaries draining sedimentary rocks in the High Andes and Mountain front have significantly higher [Re]_diss_ (3.22 ± 1.82 pmol L^−1^, 1σ, n = 38) than those draining the foreland (0.70 ± 0.53 pmol L^−1^, 1σ, n = 9). For first-order catchments, [Re]_diss_ is the highest in the High Andes (0.12 to 0.48 pmol L^−1^), as compared to <0.15 pmol L^−1^ in the Mountain front and foreland. In contrast, no significant change in [Re]_diss_ downstream is observed for nested catchments, although the average concentration at WAY (3.76 ± 0.76 pmol L^−1^, 1σ, n = 6) is slightly higher than at the SP site (2.59 ± 0.44 pmol L^−1^, 1σ, n = 7). We note that in March 2019, [Re]_diss_ was higher (4.22 pmol L^−1^) at SP than the average [Re]_diss_ of time-series measurements from the 2010 to 2011 period, perhaps related to a large landslide in this catchment in the intervening period. At the foreland-floodplain site (CICRA), [Re]_diss_ = 3.69 ± 0.98 pmol L^−1^ (1σ, n = 9). For the main tributaries of the Madre de Dios, we observe the highest [Re]_diss_ in the Manú (1.5 to 9.5 pmol L^−1^), lowest [Re]_diss_ in the Chiribi and Colorado rivers (0.5 to 2.2 pmol L^−1^), and intermediate [Re]_diss_ in the Alto Madre de Dios (3.4 to 4.7 pmol L^−1^), Inambari (3.3 pmol L^−1^) and the Tambopata (2.3 pmol L^−1^).

### Temporal Variability of Dissolved Re Concentrations.

In the four nested-catchments, [Re]_diss_ generally decreases with increasing instantaneous runoff, Q, except for the high-altitude Andean site (WAY) where [Re]_diss_ shows no clear relationship with runoff (Fig. 1*D*). The relationship can be modeled using a power law [Re]_diss_ = a × Q^b^, with a and b as the two fitted constants. For our samples, we observe a decrease in the b value from the Andes (–0.09 ± 0.12 in WAY, –0.15 ± 0.08 in SP; 1σ) to the mountain front (–0.40 ± 0.06 in MLC) and the foreland-floodplain (–0.46 ± 0.08 in CICRA), indicating a more chemostatic behavior of Re in the Andes, and more dilutional behavior in the foreland-floodplain. This decrease in the b value with elevation is similar to that observed for most major elements (29).

### Re Concentrations in Solids.

**Fig. 2.**
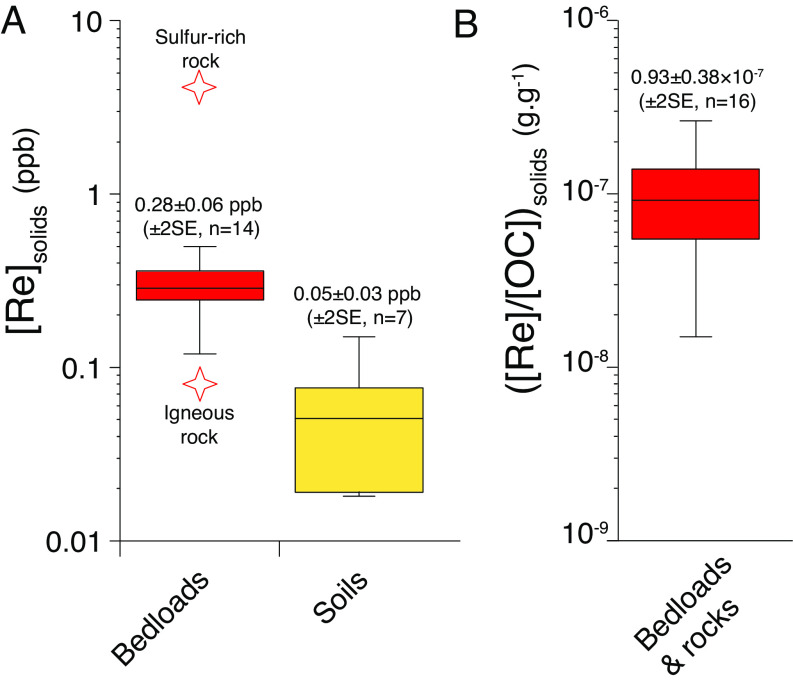
(*A*) Rhenium concentration in solids ([Re]_solids_): rock samples (red stars), bedloads (red box) and soils (yellow box). The middle line in boxes is the average value, upper and lower box limits correspond to the ±2 SE values, the upper and higher bars represent extreme data points. (*B*) Average ±2 SE and extreme values of the [Re]/[OC] ratio in bedloads and rocks.

The Re concentration in bedloads ([Re]_BM_) from Andean rivers draining shales ranges from 0.12 to 0.50 ppb with an average of 0.28 ± 0.06 ppb (±2 SE, n = 14; Fig. 2). These are higher than the Re concentrations in Andean soil samples (39) collected close the SP site 0.07 ± 0.07 ppb (±2 SE, n = 3) and in the foreland-floodplain 0.04 ± 0.01 ppb (±2 SE, n = 4). The [Re]_BM_ is slightly higher in WAY (0.31 to 0.36 ppb) compared to SP (0.23 to 0.26 ppb). Two rock samples were analyzed: [Re] of the OC_petro_ and sulfur-rich sedimentary rock sample is almost 100 times higher (4.61 ppb) than the igneous rock (0.06 ppb). The [Re]/[OC] ratio in bedloads and rocks vary from 0.27 × 10^−7^ to 2.65 × 10^−7^ g g^−1^ with an average of 0.93 ± 0.38 × 10^−7^ g g^−1^ (±2 SE, n = 16; Fig. 2). The average [Re]/[OC] for the Kosñipata stream bedload (WAY and SP sites) is 0.80 ± 0.12 × 10^−7^ g g^−1^ (±2 SE, n = 5). This [Re]/[OC] ratio value is similar to bedload from New Zealand Southern Alps (4) but lower than bedloads from Taiwan (17), the Yamuna River (18) and the Mackenzie River (20).

## Discussion

Our aim is to determine the rates and controls of OW of OC_petro_ across the transition from the Andes Mountains to the Amazon floodplain in the Madre de Dios basin. To do so, we need to characterize the source, behavior, and fluxes of dissolved Re. Following previous studies (4, 17, 19, 20, 40), the OC_petro_ oxidation yield (*J*_OCpetro-ox_, gC km^−2^ y^−1^) can be estimated from:

[1] JOCpetro-ox=JRe×fC×OC/Resolids×1-fgraphite,

where “J_Re_” is the dissolved Re yield (in g km^−2^ y^−1^), the product of the Re concentration and runoff (J_Re_ = [Re]_diss_ × Q), while “([OC]/[Re])_solids_” is the OC/Re ratio in sedimentary rocks being weathered (in g g^−1^), “*f*_C_” is the fraction of dissolved Re deriving from the oxidation of OC_petro_ and “*f*_graphite_” is the proportion of graphite in the rocks that may be resilient to OW (14).

### Dissolved Re Source and Yield.

To determine the OC_petro_ oxidation flux using the Re proxy, we must first quantify the proportion of dissolved rhenium derived from OC_petro_ oxidation relative to other potential Re sources (rainwater, carbonates, sulfides, silicates, carbonates; ref. 18). Following the approach developed in ref. 20, we use the [Re]/[SO_4_^*^] and [Re]/[Na^*^] ratios to do so ([Na^*^] and [SO_4_^*^] are concentrations corrected for atmospheric-derived contributions). In general, sulfides have low Re/SO_4_ (41) and silicates low Re/Na relative to OC_petro_ (20), and both Na and SO_4_ are conservative soluble species in the Madre de Dios catchment (34, 35, 38). In analogy with studies on silicate and carbonate weathering, here we use local constraints on the end member compositions, using the combination of water samples from first-order catchments and tributaries, combined with river sediments and rocks (23, 42) (*Materials and Methods*).

**Fig. 3.**
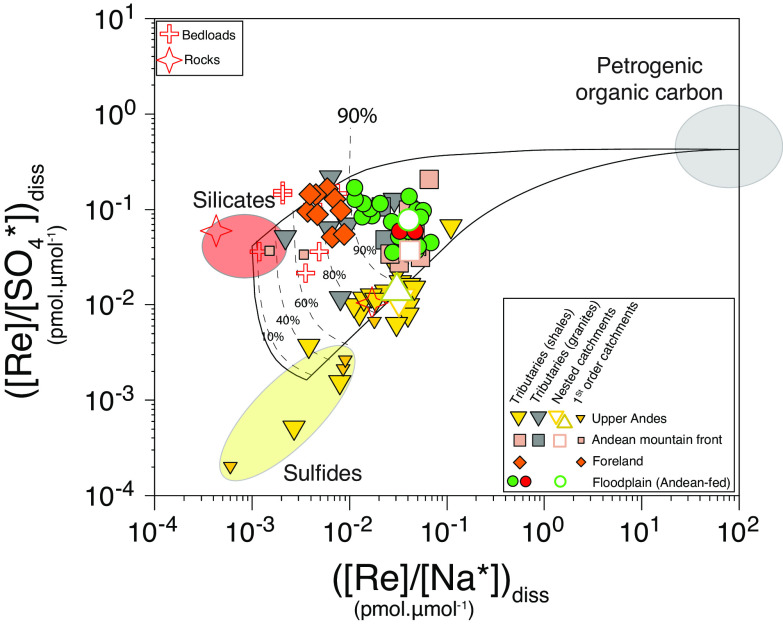
Rhenium to sodium, [Re]/[Na*], and rhenium to sulfate, [Re]/[SO_4_*], ratios (pmol μmol^−1^) for river waters from this study (High Andes = triangles; Andean Mountain front = square; Foreland = diamond; circles = Foreland-Floodplains). For the four nested-catchments, only the average values are represented. The red crosses correspond to the river sediments (bedloads) and bedrocks from the Kosñipata River basin. The shaded ovals show the ranges of elemental ratios associated with the rock weathering end-members (sulfides, silicates, and OC_petro_). The lines correspond to the mixing proportions between the rock weathering end-members, with the average proportion of Re derived from OC_petro_ weathering shown (in %).

We find that small Upper Andes tributaries draining shales have a large range of [Re]/[SO_4_^*^] and [Re]/[Na^*^] ratios that can be interpreted as a mixing trend between sulfides and OC_petro_ weathering, with limited Re contribution from silicate weathering (Fig. 3). In contrast, Andean tributaries draining granites, and rivers draining the foreland, have ratios that can be mostly explained by a mixture between silicates and OC_petro_ (Fig. 3). A mixing analysis (*Materials and Methods*) shows that the fraction of dissolved Re derived from OC_petro_ oxidation, *f*_c_ (Eq. **1**), is >0.75 in the Andes (sites WAY and SP) and increases downstream to >0.90. Seasonal variability of the Re source is small but significant (less than 20% variability), with higher proportion of Re derived from OC_petro_ oxidation in the Andes at higher runoff. This could reflect the larger contribution of shallow groundwaters from the unsaturated zone above the sulfide OW front (19). The fraction of Re derived from sulfide OW decreases from ~0.15 to 0.25 in the Andes (WAY and SP) to <0.05 in the foreland-floodplain (CICRA). The fraction of Re derived from silicate weathering is generally <0.05 but reaches ~0.2 for rivers draining only the foreland (e.g., Rio Los Amigos). Altogether, these data show that in the Madre de Dios basin, the majority of dissolved Re is derived from OC_petro_ oxidation, confirming observations in other catchments where sedimentary rocks dominate the geology (19, 20, 43).

Calculation of dissolved Re yield (J_Re_) can be done in several ways, i.e., using average [Re]_diss_ (4, 17, 18, 44), discharge-weighted average [Re]_diss_ (41) or rating curve-derived [Re]_diss_ (19) and annual or instantaneous water discharge estimates. Here, these methods return similar results (*Materials and Methods* and Dataset S3). Therefore, we take advantage of paired [Re]_diss_ and discharge measurements (at the four nested catchment sites and at the main tributaries) and use discharge-weighted average [Re]_diss_ and annual water discharge to derive dissolved Re yield. For the main tributaries, the highest J_Re_ is in the Manú River catchment (2.0^+0.9^/_−0.7_ g km^−2^ y^−1^) and the lowest is in the Las Piedras river (0.3 ± 0.1 g km^−2^ y^−1^). For the nested catchments, the Re yield is 2.1 ± 1.0 g km^−2^ y^−1^ and 1.3 ± 0.2 g km^−2^ y^−1^ for WAY and SP sites, respectively, and 1.6 ± 0.4 g km^−2^ y^−1^ for the foreland-floodplain (CICRA) site. Although there is a slight decrease with elevation, the main result is that the specific Re yield of the Rio Alto Madre de Dios does not change, within uncertainty, when the Rio Alto Madre de Dios (Andes) reaches the foreland-floodplain.

### Rates and Control of OC_petro_ Oxidative Weathering in the Andes.

We compute the OC_petro_ oxidative weathering rate (Eq. **1**) using the parameter values defined above (Dataset S3). As we have no constraint on *f*_graphite_ in this setting, we do not account for this term here. To estimate the uncertainties, we use Monte Carlo simulations with 10,000 resolutions of the equation with distribution sampling of values within the errors of each parameter (4). The estimated OC_petro_ oxidative weathering fluxes based on dissolved Re yield are 16.7 ^+11.9^/_−8.8_ tC km^−2^ y^−1^ for the WAY site and 11.2^+4.5^/_−2.8_ tC km^−2^ y^−1^ for the SP site. The lower uncertainty on the J_OCpetro-ox_ for SP is due to a higher precision on the water flux at this site (33).

It has been proposed that physical erosion rate is a major control on the rate of OW (3, 8), with a potential role for landslide erosion supplying fresh mineral surfaces. The present-day erosion rate of the Rio Kosñipata is high, with values between 1,200 and 3,500 t km^−2^ y^−1^ (11) and frequent landslides (45). The J_OCpetro-ox_ for the Kosñipata is much higher than in the Mackenzie (0.45 to 1.01 tC km^−2^ y^−1^) and the Swiss Alps (3.6 to 5.7 tC km^−2^ y^−1^) characterized by lower erosion rates, and more similar to rivers in New Zealand and Taiwan (4 to 30 tC km^−2^ y^−1^), which have high erosion rates (3). Results from this study thus confirm the general control of erosion rate on the rate of OC_petro_ oxidation (3), though other factors can also play an important role (3, 46). The unweathered solid OC_petro_ export at the SP site has been determined previously to be 16.1 ± 1.4 tC km^−2^ y^−1^ using sediment samples collected across the same time period (11). These fluxes imply that 41 ± 10% of the total bedrock OC_petro_ is oxidized in the Andes, and the remainder is exported downstream. This OC_petro_ weathering intensity value is slightly lower than in the Mackenzie River basin (50%), similar to the Swiss Alps (19) and higher than in Taiwanese rivers (<20%) (17).

Our estimate of the flux of OC_petro_ oxidation in the Rio Kosñipata is an attempt to quantify the CO_2_ emission from weathering of OC_petro_ in the Andes. This estimate can also be compared with other carbon fluxes relevant to the long-term carbon cycle in the Andes in the Kosñipata catchment. In the SP catchment, the rate of biospheric organic carbon (OC_bio_) export in river suspended sediments is 12.6 ± 0.4 tC km^−2^ y^−1^ (11), which is slightly higher than the OC_petro_ oxidative weathering yield. At the same site, the yield of carbon release associated with carbonate weathering by sulfuric acid is ~6 tC km^−2^ y^−1^ (23), 45% lower than the rate of CO_2_ release by OC_petro_ oxidation. Together, the OW of reduced phases (OC_petro_ and sulfides) in Andean sedimentary rocks releases about 17 tC km^−2^ y^−1^, confirming that OW of sedimentary rocks in mountain belts is a large source of CO_2_ to the atmosphere.

### Oxidative Weathering in the Foreland and in the Andean-Fed Floodplains.

Once the OC_petro_ that survived OW in the Andes has reached the foreland and the floodplains, it can undergo further OW during transport and deposition in the floodplains (10). It has been suggested that this process can be a large source of CO_2_ to the atmosphere in the neighboring Beni catchment (10) and in the Ganges floodplain (14). Here, we take several approaches to isolate the weathering signal from the foreland (here defined as <500 m and not Andean-fed) versus the Andean-fed floodplain (<500 m low relief adjacent to active river channels where modern Andean-derived sediments are exchanged). First, we assess the OC_petro_ weathering flux in the foreland by using data from the Las Piedras river catchment, since it drains the foreland exclusively with no Andean contribution. The [Re]_diss_ concentration at Las Piedras (1.24 ± 0.35 pmol L^−1^, 2 SE) is low like the two other medium-size foreland rivers, the Blanco (0.36 ± 0.06 pmol L^−1^) and the Los Amigos (0.50 ± 0.16 pmol L^−1^). The [Re]/[OC] ratio of bedrock in Las Piedras can be reasonably assumed to be equivalent to the Andean-derived bedload from the Rio Kosñipata, given that the foreland-floodplain region is predominantly underlain by ancient Andean-derived sediments (23). Using this assumption, we calculate a J_OCpetro-ox_ of 1.9^+1.2^/_−0.8_ tC km^−2^ y^−1^, which is about six times lower than in the Andes and similar to the rate measured in the Mackenzie River basin (20).

**Fig. 4.**
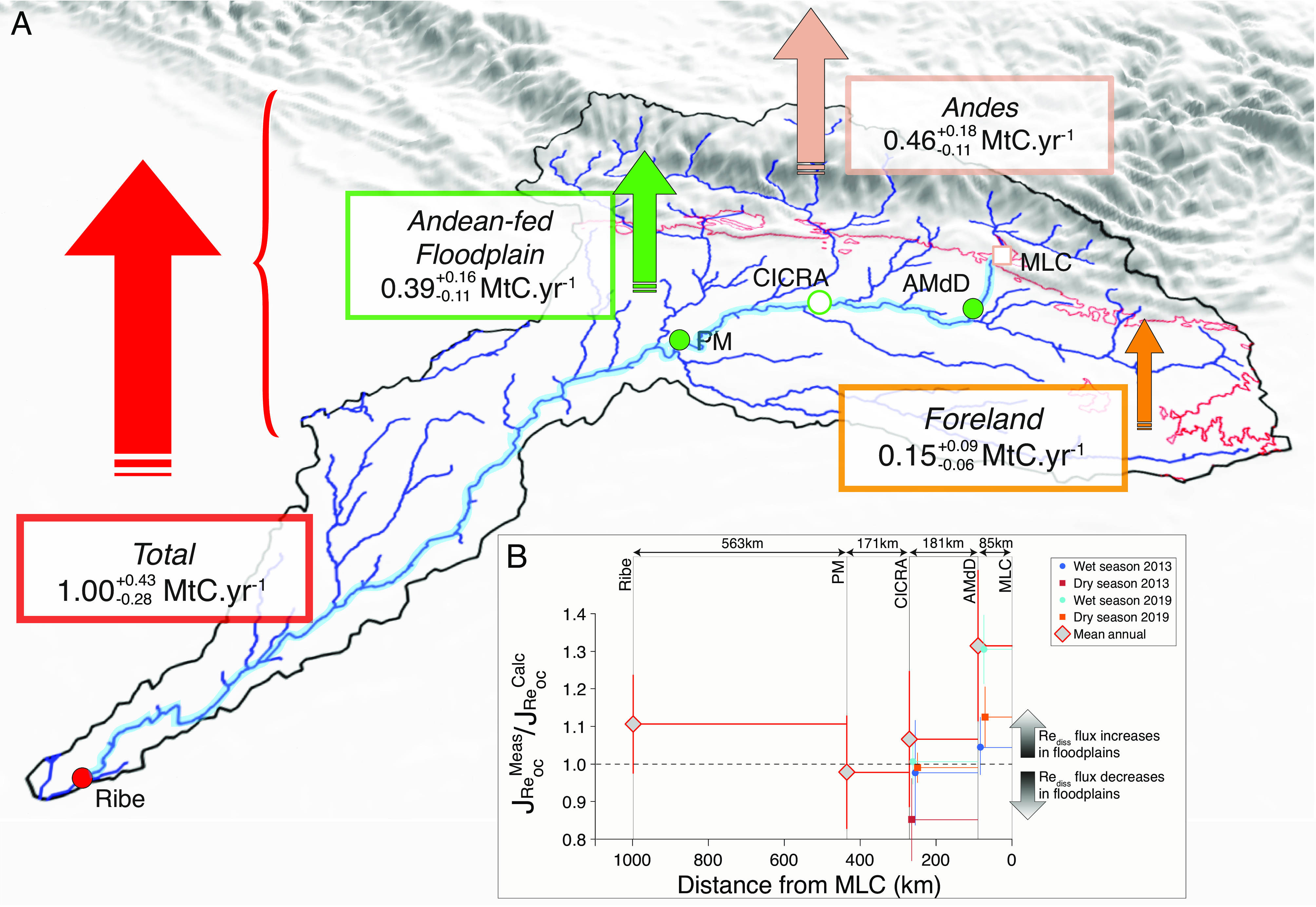
(*A*) Estimated net contribution (vertical arrows) of each geomorphic zone to the total flux of OC_petro_ oxidation and associated CO_2_ release at the scale of the whole Madre de Dios River basin. MLC = Mountain Front, AMdD = Alto Madre de Dios, PM = Madre de Dios at Puerto Maldonado, Ribe = Madre de Dios at Riberalta. The red squiggly line corresponds to the 500-m elevation contour. (*B*) Ratio between the measured dissolved rhenium flux derived from OC_petro_ oxidation (J_Re-OC_^meas^) and the predicted flux (J_Re-OC_^calc^) using the tributary mixing model (*SI Appendix*), as a function of the channel distance from MLC. A value >1 can be interpreted as OC_petro_ oxidation in the floodplain. Vertical bars represent the uncertainties. The studied floodplain transect corresponds to the channel highlighted in light blue on panel *A*.

To estimate the OC_petro_ weathering flux in the floodplains, we attempt a mass-balance at the scale of the whole Madre de Dios catchment (118,459 km^2^ at El Sena station near Riberalta) (37). Assuming the *J*_OCpetro-ox_ from SP (Upper Andes) is representative of the Andean area (40,868 km^2^; defined as <500 m) of the Madre de Dios catchment, we calculate a total Andean *J*_OCpetro-ox_ of 0.46^+0.18^/_−0.11_ MtC y^−1^. This assumption is justified as the SP catchment has similar erosion rates as the inferred Andean erosion rate for the whole Madre de Dios River (37). The contribution from foreland weathering (<500 m; 77,591 km^2^) can be estimated using the *J*_OCpetro-ox_ from the Las Piedras River (19,630 km^2^) as 0.15^+0.09^/_−0.06_ MtC y^−1^. The total *J*_OCpetro-ox_ at the mouth of the Madre de Dios is 1.00^+0.43^/_−0.28_ MtC y^−1^. Hence, the contribution of Andes + Foreland is lower than the total *J*_OCpetro-ox_ of the Madre de Dios at its mouth by 0.39^+0.16^/_−0.11_ MtC y^−1^, which we attribute to weathering in the floodplain. This mass-balance indicates that ~46% of the total OC_petro_ oxidation takes place in the Andes, ~40% in the Andean-fed floodplains, and ~14% in the (non Andean-fed) foreland-lowlands (Fig. 4*A*).

The deposition and weathering of sediments in the floodplain constitutes a different mechanism compared to upland weathering (29, 47). Sediment and water exchange and storage during floodplain transit by rivers can result in long sediment residence times (48), and floodplain structure influences water flux and redox state, which could facilitate OW (7). In addition, the warmer climatic conditions in the floodplain relative to the Andes could be important in setting reaction rates (21), while the length of floodplains and their channel migration rates (49) could also be important controlling variables. Scaled laboratory experiments have been used to suggest that in situ oxidation during transport within river channel is small (50) meaning reactions in sediment stores of floodplains are likely to be of most importance (51).

Our paired instantaneous [Re]_diss_ and discharge measurements from the same sampling trips (in 2013 and 2019, both wet and dry seasons) covering both upstream and downstream floodplain sections constrain the location of OC_petro_ oxidation reactions in the floodplains between MLC and CICRA (Fig. 4*B*). The ~85-km long section of the river along the course of the Alto Madre de Dios floodplain, between the Mountain Front (MLC) and the confluence with the Rio Manú, is mostly braided and multichannel, with mobile channel bars comprised of sand and fine gravel. Measurements show a significant increase in J_Re_ (Re flux) over this river reach. Water isotopes indicate lack of significant water, and therefore likely Re, contribution from tributaries over this distance (*Materials and Methods*). Hence, we attribute this J_Re_ increase (from 4 to 30%, Fig. 4*B*) to weathering of OC_petro_ from Andean sediments transiting in the floodplains (*SI Appendix*). The same calculation can be done using mean annual Re flux, but with a larger uncertainty. We calculate that the mean annual J_Re_ increases by 31 ± 20% which corresponds to a flux of CO_2_ release through OC_petro_ oxidation in the floodplain reach between MLC (Mountain front) and AMdD of 0.03 ± 0.01 MtC y^−1^. In comparison, the CO_2_ release through OC_petro_ oxidation in the Andean part of the Alto Madre De Dios catchment is 0.08 ± 0.02 MtC y^−1^. As the solid OC_petro_ load at MLC is 0.12 ± 0.01 MtC y^−1^ (ref. 33, assuming similar solid OC_petro_ yield as at SP), this would suggest that ~25% of Andean-derived solid OC_petro_ is oxidized before reaching the confluence with the Rio Manú over a floodplain length of 85 km.

In contrast, in the section between AMdD and CICRA (~180 km long), where a larger, single channel meanders through the floodplain, we observe no significant Re flux increase in wet seasons (2013 and 2019) and dry season 2019. For the dry season in 2013, we even observe a small decrease in Re yield along the section (Fig. 4*B*). Similarly, using mean annual J_Re_, we calculate no significant increase in OC_petro_ oxidation, within uncertainties (Fig. 4*B* and *SI Appendix*), in this section nor in the CICRA-PM section (~170 km long). Over the transit from MLC to CICRA, the isotopic composition of sulfate suggests minimal sulfate reduction (36), suggesting that oxygen is available for OW weathering along this river reach. In addition, the warm tropical climate could drive higher reaction rates and OC_petro_ oxidation (21). Therefore, the apparent limited floodplain weathering could reflect an exhaustion of reactive OC_petro_ supplied from Andean erosion upstream. Regardless of the mechanisms at play, the Rio Madre de Dios example shows that high rates of OC_petro_ oxidation in the mountain headwaters can be matched by OW in the foreland (Fig. 4). A large proportion of floodplain weathering appears to happen over a relatively short-length scale (~<85 km) after exiting the Andes.

Finally, for the section furthest downstream, PM-Ribe (~560 km long), calculation using mean annual J_Re_ shows a 10% increase in Re flux. This may reflect OW of reactive OC_petro_ supplied by the Inambari and/or the Tambopata, in combination with a longer time sediments spend in this long floodplain section (e.g., see ref. 48). Yet, this observation should be interpreted with caution due to the large uncertainty (±13%) associated with our calculation. Additional work on this part of the catchment is necessary to refine these calculations.

## Implications for the Long-Term Carbon Cycle

Previous studies on the Amazon River found significant CO_2_ release from OC_petro_ oxidation during the transit of fluvial sediments in the Madeira floodplain (10, 11). They found that the solid OC_petro_ flux from the Madeira at the confluence with the Amazon is much lower than the solid OC_petro_ supplied by the Beni at the mountain front and transported through the plain. They estimated a CO_2_ emission of 0.50 MtC y^−1^ across the Rio Beni floodplain. Our Re-based estimate of OC_petro_ oxidation during the transit of Madre de Dios sediments in the floodplain (0.39^+0.16^/_−0.11_ MtC y^−1^) is of similar magnitude. The total CO_2_ emission from OC_petro_ oxidation in the whole Madre de Dios catchment (1.00^+0.43^/_−0.28_ MtC y^−1^) is more than twice the silicate weathering CO_2_ drawdown (0.43^+0.22^/_−0.09_ MtC y^−1^; ref. 52). Considering that sulfide oxidation also contributes to the carbonate weathering flux (23), the net CO_2_ balance during weathering in the Madre de Dios catchment appears to be tipped firmly toward being a CO_2_ source (3).

Our findings from the Rio Madre de Dios catchment allow us to postulate a broader role for floodplain weathering in enhancing the CO_2_ release by OC_petro_ oxidation. Uplift and exhumation of sedimentary rocks in a mountain range can increase the supply of OC_petro_ to an oxygenated weathering zone (17, 21) and increase the rates of OC_petro_ oxidation and CO_2_ release, as we observe in the Andes (Fig. 4). However, the overall weathering intensity can be low, with only ~20 to 50% of OC_petro_ oxidized, meaning there is further potential for CO_2_ release. On high standing mountain islands, floodplains are short. For instance, in Taiwan rivers have floodplains <40 km long, and many are shorter than 5 km, and there unweathered OC_petro_ is reburied offshore (16). However, if the tectonic setting permits the growth of a larger continental floodplain, additional OC_petro_ oxidation is very likely to occur even if just ~100 km in length based on our findings (Fig. 4). In this case, only extremely refractory OC_petro_ escapes oxidation (10). While future work will need to establish how temperature and O_2_-supply impact OC_petro_ oxidation rates in floodplains (7, 21), our results suggest that the formation of floodplain adjacent to a mountain range allows more complete OC_petro_ oxidation during sediment transit. Over multimillion year timescales, the growth and waning of riverine floodplains and continental sediment storage (27) could thus act as a powerful carbon cycle modifier throughout Earth’s history.

## Materials and Methods

The materials and methods are summarized here; further details are provided in *SI Appendix*. All data used in this study are reported in Datasets S1–S5.

### Sample Collection and Discharge Measurements.

Major cation and anion concentration data of samples from 2010 to 2013 are from refs. 23 and 29. Detailed information on the sampling protocol can be found in those studies. In summary here, for the four main nested catchment sites (WAY, SP, MLC, and CICRA, Fig. 1), time-series samples were collected between 2010 and 2011 using a clean polypropylene (PP) bottle and filtered on site with a 0.2-μm porosity nylon filter (29). At these sites, water discharge was measured at the same time as sampling by monitoring water levels manually and converting to discharge using a rating curve. At the SP site, river level was monitored with a water level logger that recorded river level measurements every 15 min (33). At other localities, samples were collected on fieldtrips in 2012, 2013, 2016, and 2019 (Dataset S1). Water samples were collected from the river surface using a clean bucket and transferred to 10- or 20-L plastic bags before filtration. Samples were filtered within 24 to 48 h of collection with 0.2-μm porosity polyethersulfone (PES) filters. The discharge (“Q”) at CICRA was measured during each sampling trip using an Acoustic Doppler Current Profiler (ADCP, RD1 Sentinel GED154 in March 2013 and SonTek M9 in August 2013 and in March and May 2019). In 2019, the discharge of the Alto Madre de Dios, Rio Manú, Chiribi and Colorado were also measured by ADCP. All discharge measurements are from refs. 34 and 35.

### Re Concentration Measurements in Water and Sediments.

The dissolved Re concentrations were measured following the same protocol as described in ref. 19. Briefly, dissolved Re concentrations ([Re]_diss_) were measured by direct calibration against a set of seven standards with varying Re abundances and similar matrixes to river water, by quadrupole inductively coupled plasma mass spectrometry (Q-ICP-MS, Agilent Technologies 7900). Calibration standards and samples were doped with 0.025 mg/L concentration of internal standard Tb and Bi to correct for instrumental drift and matrix effects. Accuracy and precision of the measurements were assessed by repeated measurements of various riverine standard reference materials, in particular reference materials SLRS-5 and SLRS-6 at various dilutions. The standards confirmed better than 10% accuracy and precision. For sediment samples, the rhenium concentrations were determined using the method in ref. 53. A mass of 0.2 to 0.5 g was digested using a mixture of 3 mL 27M HF and 3 mL 16M HNO_3_ for 24 to 48 h at 120 °C on a hot plate. Digested solutions were processed through AG1-X8 resin to separate Re from the rest of the matrix. Rhenium concentrations were then measured with a Neptune MC ICP-MS at Durham University.

### Dissolved Re Yield Calculations.

Several methods can be used to determine dissolved ion yields depending on the number of paired water discharge (Q) and concentration measurements, their frequency, and the behavior of the element of interest in relation with discharge (54). Previous studies with large datasets of Re concentration and discharge (19, 20) have used a rating curve approach to quantify dissolved yields, by fitting power law functions to the trends in the data and using this relationship to predict daily discharge value and annual fluxes. Other studies have used discharge-weighted average concentration (41) or average concentration of several measurements (4, 17, 18, 44) combined with annual water discharge estimates.

The four nested catchments in our dataset have between five and nine Q and [Re]_diss_ data pairs for each catchment (Fig. 1*C*). For the large tributaries (Manú, Colorado, Chiribi, Alto Madre de Dios), our dataset includes four paired Q and [Re]_diss_ measurements each. For all the sites, we use three different methods to calculate the Re yield and uncertainties: i) mean [Re]_diss_ ± SE multiplied by annual discharge; ii) discharge-weighted mean [Re]_diss_ ± SE multiplied by annual discharge; and iii) average of measured instantaneous Re fluxes, J_Re_ ± SE. The advantage of the first method is that it includes more [Re]_diss_ measurements (since the instantaneous discharge was not measured for all samples). The disadvantage is that there is a potential bias toward high concentration by not weighting to discharge. The third method is useful if annual discharge is not known; however, it has the disadvantage of being less accurate because instantaneous fluxes vary more than instantaneous concentrations (54). Discharge values and uncertainties are reported in Dataset S3. For the WAY and SP catchments, we use annual discharge values from ref. 33, determined over the year 2010. In addition, we use average annual discharge values dataset for the main tributaries of the Madre de Dios that were determined by water balance over the period 1968 to 1982 (55). They calculate a total discharge at the mouth of the Madre de Dios (Riberalta) of 6,369 m^3^ s^−1^ which are very close (only 12% higher) to the discharge measured during the 2002 to 2011 period (5,661 m^3^ s^−1^; ref. 37). For CICRA, the discharge can be estimated by adding the annual discharge values of the Manú, Alto Madre de Dios, Colorado from ref. 55 and considering that the Rio Chiribi contributes 7.5% of the total discharge in CICRA (Dataset S5). This gives a value of 2,165 m^3^ s^−1^. For instantaneous discharge of the main tributaries (Alto Madre de Dios, Manú, Chiribi and Colorado) that were measured or calculated during the 2013 and 2019 sampling trip, we use values from previous studies (34, 35).

Calculation of uncertainty is done by a Monte Carlo simulation (run 10,000 times for each watershed). As the calculated distributions follow skewed rather than normal distributions, we report the median value (50th percentile) with the uncertainty range defined by the 16th and 84th percentiles (equivalent to 68% of the entire population; *SI Appendix*). Comparison between the various methods shows good agreement between the different methods for the different sites within uncertainties (Dataset S3). The third method gives larger uncertainty compared to methods 1 and 2. This could reflect the lower number of samples used, the uncertainty on the discharge measurement, or an imbalance between the discharge during the 1968 to 1982 period and the 2010 to 2020 period. When quantifying OC_petro_ oxidation fluxes from the dissolved Re flux for all sites, we use the second method (discharge-weighted [Re]_diss_) when possible and the first method (mean [Re]_diss_) otherwise.

### Source Partitioning of Dissolved Rhenium.

To quantify the OC_petro_ oxidation flux using dissolved Re, we need to: i) correct for Re inputs from precipitation and/or atmospheric deposition; ii) quantify the Re input from non-OC_petro_ sources (i.e., sulfide, carbonate, and silicate minerals). Solutes in precipitation can come from dissolution of sea salts, dust or biogenic particles. The [Re]/[Cl] ratio (~7.5 × 10^−5^ pmol μmol^−1^) of the ocean is very low (56) compared to the [Re]/[Cl] of rivers from the Madre de Dios. The [Cl]_diss_ in rivers from this study are also low, indicating that the proportion of Re derived from sea salts is very small. While we have only one rainwater sample from the upper Andes, its [Re]_diss_ was below detection limit of the measurement session (<0.05 pmol L^−1^), supporting this conclusion. Among all the samples, the lowest measured [Re]_diss_ and [Re]/[Cl] ratio correspond to a lysimeter sample in the Foreland (PER19-38) with values of 0.009 pmol L^−1^ and 1.4 × 10^−3^ pmol μmol^−1^, respectively. The [Re]/[Cl] ratio of this sample is ~20 times higher than the [Re]/[Cl] ratio of the ocean. The major element concentration for this sample is in the range of the average composition of rainwater from the Andes from ref. 29. Hence, we use this sample as representative of the maximum Re/Cl ratio of rainwater. For calculating the proportion of Re derived from each source we used the following mass-balance. For the contribution of the rain:

[2] $${\left[Re\right]}_{rain}={\left(\frac{Re}{Cl}\right)}_{rain}\times {\left[Cl\right]}_{cycl},$$

where (Re/Cl)_rain_ is the proposed elemental ratio between Re and the Cl in the rain (1.4 × 10^−3^ pmol μmol^−1^) and [Cl]_cycl_ is the cyclic chlorine concentration. Since the marine evaporite contribution is small or negligible in the Madre de Dios catchment (23, 29), we consider that [Cl]_cycl_ = [Cl]_riv_. The concentration of any element X corrected for rainwater and evaporite inputs is referred as “[X*]”. We find that the proportion of riverine Re deriving from rainfall is negligible, being generally less than 0.5%, with a maximum of 6%. For SO_4_ rain concentration, we use a (SO_4_/Cl) value of 0.53 corresponding to the median value of precipitation data from ref. 29. This value is only slightly lower than the lowest (SO_4_/Cl) measured in our dataset (sample PER19-38, value of 0.60).

We then move to quantify the proportion of dissolved rhenium derived from OC_petro_ oxidation relative to other potential Re sources (sulfides, silicates, carbonates). Previous work has suggested carbonates are not a major source of dissolved Re (18). If the Re/Ca of carbonates is ~5 × 10^−5^ pmol mol^−1^ (18), <1% of total dissolved Re in the studied rivers here can be accounted for by carbonate weathering (a maximum proportion using this Re/Ca ratio and assuming all dissolved Ca in the Madre de Dios catchments is derived from carbonates). We therefore follow the approach developed in ref. 20, that uses the (Re/SO_4_)* and (Re/Na)* ratio to characterized Re input from sulfides, silicates and OC_petro_.

Sulfides have low Re/S (41) and silicates low Re/Na relative to OC_petro_ (20) and both Na and SO_4_ are conservative soluble species in the Madre de Dios catchment (34, 35, 38). Assuming that all the [SO_4_^2−^*] is derived from pyrite oxidation (i.e., no evaporite contribution, ref. 23), we estimate the Re concentration derived from sulfide oxidation:

[3] $${\left[Re\right]}_{diss.sulfides}={\left(\frac{Re}{{SO}_{4}^{2-}}\right)}_{sulfides}\times \left[{\mathrm{SO}}_{4}^{2-}*\right],$$

where (Re/SO_4_^2−^)_sulfides_ is the sulfide composition. Then we can determine the concentration of Re deriving from silicate weathering ([Re]_diss_._sil_) using sodium and assuming that all the [Na*] is derived from silicate weathering:

[4] $${\left[Re\right]}_{diss.sil}={\left(\frac{Re}{Na}\right)}_{silicates}\times \left[\mathrm{Na}*\right],$$

where (Re/Na)_sil_ is the silicate signature. Then, we attribute the excess Re to the oxidation of OC_petro_, calculated as:

[5] Rediss.OC=Re∗-Rediss.sulfides-Rediss.sil.

In analogy with studies on silicate and carbonate weathering, we use small tributaries (e.g., refs. 57 and 58) and/or sediment composition (23, 42) to constrain local weathering end-members [Re]/[SO_4_] and [Re]/[Na] ratio values (20). Small Andean tributaries draining sulfur-rich metasedimentary rocks display two orders of magnitude variability in [Re]/[SO_4_^*^] and [Re]/[Na^*^] ratios and are positively correlated. Several Andean rivers with low [Re]/[SO_4_^*^] values (Fig. 3) have similar composition to the median Re/S values of pyrite from the literature (1.8^+4.2^/_−1.7_ × 10^−3^ pmol μmol^−1^, ref. 41) with the lowest [Re]/[SO_4_^2-^] ratio corresponding to a lysimeter sample (PER19-97) in the riparian area of a small catchment ([Re]*/[SO_4_^2−^]* = 2.2 × 10^−4^ pmol μmol^−1^) and a river sample (r2400) from another small catchment in the Andes ([Re]*/[SO_4_^2-^]* = 4.8 × 10^−4^ pmol μmol^−1^). They both have high SO_4_ concentration indicating a high rate of sulfide oxidation. The [Re]/[SO_4_^*^] ratio of these samples is similar to the sulfide-oxidation rich sample in the Mackenzie basin (20). Hence, this suggests that the Re and SO_4_ composition of these rivers is dominated by sulfide oxidation of pyrite and that they can be used as end-member values for (Re/SO_4_)_sulfides_. The lowest (Re/SO_4_)* value (2.2 × 10^−4^ pmol μmol^−1^) measured here could represent the “purest” sulfide oxidation end-member and higher [Re]/[SO_4_^*^] for other rivers would be explained by a small contribution of Re from rock organic carbon oxidation. Alternatively, it is possible that the Re/S from the local bedrock is variable and explains the range of [Re]/[SO_4_^*^] observed in rivers dominated by sulfide oxidation. A third possibility is that the very low [Re]/[SO_4_^*^] and [Re]/[Na^*^] of some samples is due to nonconservative behavior and removal of dissolved Re because some of these rivers have low pH values (as low as 3.5) and ReO_4_^−^ is less stable under acidic conditions (59). However, this cannot explain the low [Re]/[SO_4_^*^] value of sample PER19-3 (3.5 × 10^−3^ pmol μmol^−1^), which has pH value of 8. Considering the above discussion, we consider (Re/SO_4_)_sulfides_ ranging from 2 × 10^−4^ to 4 × 10^−3^ pmol μmol^−1^ that encompass the range of [Re]/[SO_4_^*^] of Andean rivers that we identified as typical of sulfide oxidation.

Rivers draining mostly granites (sulfide-poor and no OC_petro_) have low Re/Na but an order of magnitude higher [Re]/[SO_4_] ratios relative to Andean tributaries draining shales (Fig. 3). Interestingly, rivers and lysimeter samples from the foreland have similar composition as rivers draining granites. For Foreland rivers, especially at low elevations, deeply weathered soils and high weathering intensity probably leads to almost complete oxidation of pyrite due to its fast kinetics, and therefore we expect the chemical composition of those samples to be less influenced by OW of pyrite than Andean rivers. Altogether, rivers draining granite and the Foreland have ratios that are inferred to reflect mostly mixing between weathering of silicates and weathering of OC_rock_. These observations are supported by river bed sediment samples. For Andean rivers, the [Re]/[Na] ratio of river bed sediments is similar to the low [Re]/[Na^*^] of small tributaries draining granites (lowest value is 2.2 × 10^−3^ pmol μmol^−1^ for sample PER19-26) and of samples from small first-order catchments draining the Foreland (lowest value is 1.5 × 10^−3^ pmol μmol^−1^ for sample PER19-35). In addition, one rock sample, corresponding to an igneous rock (a granophyre), has a very low [Re]/[Na] ratio of 4.3 × 10^−4^ pmol μmol^−1^, similar to the crystalline rock endmember [Re]/[Na] ratio value in the Himalaya (2 × 10^−4^ pmol μmol^−1^, ref. 18). Hence, we consider here a [Re]/[Na] ratio between 4 × 10^−4^ and 2 × 10^−3^ pmol μmol^−1^ for silicates, which spans the range of lowest [Re]/[Na] values from local solid and river samples.

Using Eqs. **1**–**4** and the range of above defined (Re/SO_4_)_sulfides_ and (Re/Na)_silicates_ values for our study area, we can calculate [Re]_diss.OC_ for each sample. The calculation of [Re]_diss.OC_ is done by a Monte Carlo simulation (run 10,000 times for each river) assuming random distribution of (Re/SO_4_)_sulfides_ values between 2 × 10^−4^ and 4 × 10^−3^ pmol mol^−1^ and (Re/Na)_silicates_ values between 4 × 10^−4^ and 2 × 10^−3^ pmol μmol^−1^ (*SI Appendix* and Dataset S4). Fractions of dissolved Re derived from OC_petro_ OW (*f*_c_ = [Re]_diss.OC_/[Re]_diss_) are reported in Dataset S3.

### Floodplain Mass-Balance of Dissolved Re.

The section of the Alto Madre de Dios river between MLC (mountain front) and AMdD (confluence with Manú River) has relatively minimal tributary input. Over three sampling trips, we observe an increase in the Re concentration between MLC and AMdD of 3% (March 2013), 26% (March 2019) and 12% (May 2019). There is also a systematic increase in Re/Na and Re/SO_4_ ratio. The Re concentration of sub-Andean tributaries is about 0.8 ppt, which is higher than the Re concentration at MLC and could explain part of the observed increase. The contribution of water and weathering inputs from sub-Andean-foreland tributaries can be assessed using δD and δ^18^O since tributaries (PER19-53, Rio Carbon, Rio Pini Pini) have higher δD (−54.9 to −70.2‰) and δ^18^O values (−8.2 to −10.4‰) compared to the Alto Madre de Dios at MLC (δD ~ −72.5‰ and δ^18^O ~ −10.6‰). The δD and δ^18^O of the Alto Madre de Dios do not change between MLC and AMdD, which indicates that any Re contribution from these tributaries is too small to explain the observed 26% increase in Re concentration in 2019. Therefore, we conclude that this increase is due to ongoing OC_petro_ oxidation of sediment during transit and/or within the floodplain between MLC and AMdD.

## Supplementary Material

Appendix 01 (PDF)

Dataset S01 (XLSX)

Dataset S02 (XLSX)

Dataset S03 (XLSX)

Dataset S04 (XLSX)

Dataset S05 (XLSX)

## Data Availability

All study data are included in the article and/or supporting information.
