# Supplementary material for: High rates of rock organic carbon oxidation sustained as Andean sediment transits the Amazon foreland-floodplain
Source: Proc Natl Acad Sci U S A. 2023 Sep 19;120(39):e2306343120. doi: 10.1073/pnas.2306343120 (PMC10523614; doi:10.1073/pnas.2306343120)
Supplement: Supplementary file 1 — Appendix 01 (PDF) [file pnas.2306343120.sapp.pdf]

PNAS

Supporting Information for

High rates of rock organic carbon oxidation sustained as Andean sediment transits the Amazon foreland-floodplain

Mathieu Dellinger, Robert G. Hilton, J. Jotautas Baronas, Mark A. Torres, Emily I. Burt, Kasey E. Clark, Valier Galy, Adan Julian Ccahuana Quispe, A. Joshua West

Mathieu Dellinger
Email: mathieu.dellinger@univ-smb.fr

Robert G. Hilton
Email: robert.hilton@earth.ox.ac.uk

This PDF file includes:

Supplementary text
Matlab codes used for calculation of Re sources, oxidation fluxes and uncertainties

Other supporting materials for this manuscript include the following:

Datasets S1 to S5

Supporting Information Text

Basin-wide mass balance of dissolved Re and OC_{petro} oxidation in floodplains: Below we detail the calculations for quantifying Re fluxes and OC_{petro} oxidation of Andean-derived sediments in the Madre de Dios floodplain. All the parameter values are reported in Dataset S3 and Dataset S5.

<u>Andean section, up to MLC:</u>
Our most accurate estimate of OC_{petro} oxidation yield is from the SP station at 1360 m elevation and gives a $J_{OCpetro\text{-}ox}$ of $11.2^{+4.5}/_{-2.8}$ tC km^{-2} yr^{-1}. Extrapolated to the whole Andean area of the Alto Madre de dios catchment (7551 km^2), this gives a total Andean $J_{OCpetro\text{-}ox}$ of 0.08±0.02 MtC.yr^{-1}.

At SP, the un-weathered solid OC_{petro} yield is 16.1±1.4 tC.km^{-2}.yr^{-1} (1). Extrapolated to the whole Andean area of the Alto Madre de dios catchment (7551 km^2), this gives a total un-weathered solid OC_{petro} flux of 0.12±0.01 MtC.yr^{-1}.

<u>Section between MLC and AMdD:</u>
In the section between MLC (Mountain front) and the confluence with the Rio Manu (AMdD), we can calculate the "instantaneous" and "mean annual" fluxes of Re derived from OC_{petro} oxidation upstream (MLC) and downstream (AMdD) the floodplain.

The dissolved Re flux derived from OC_{petro} oxidation (in kg yr^{-1}) at the MLC site ("$\mathrm{J}_{Re-OC}^{\mathrm{MLC}}$") is:

$$\mathrm{J}_{Re-OC}^{\mathrm{MLC}} = \frac{\mathrm{Q}^{\mathrm{MLC}} \times [\mathrm{Re}]_{\mathrm{diss}}^{\mathrm{MLC}}}{3600 \times 24 \times 365.25} \times f_C^{\mathrm{MLC}} \times 10^6 \; (Eq.1)$$

With "$\mathrm{Q}^{\mathrm{MLC}}$" and is the discharge (in m^3 s^{-1}) at the MLC site, "$[\mathrm{Re}]_{\mathrm{diss}}^{\mathrm{MLC}}$" is the dissolved Re concentration at the MLC site, "f_C^{MLC}" is the fraction of dissolved Re deriving from the oxidation of OC_{petro} and the term "$3600 \times 24 \times 365.25$" is the conversion factor from seconds to years.

The dissolved Re flux derived from OC_{petro} oxidation (in kg yr^{-1}) at the AMdD site ("$\mathrm{J}_{Re-OC}^{\mathrm{AMdD}}$") is:

$$\mathrm{J}_{Re-OC}^{\mathrm{AMdD}} = \frac{\mathrm{Q}^{\mathrm{AMdD}} \times [\mathrm{Re}]_{\mathrm{diss}}^{\mathrm{AMdD}}}{3600 \times 24 \times 365.25} \times f_C^{\mathrm{AMdD}} \times 10^6 \; (Eq.2)$$

With "$\mathrm{Q}^{\mathrm{AMdD}}$" and is the discharge (in m^3 s^{-1}) at the AMdD site, "$[\mathrm{Re}]_{\mathrm{diss}}^{\mathrm{AMdD}}$" is the dissolved Re concentration at the AMdD site, "f_C^{AMdD}" is the fraction of dissolved Re deriving from the oxidation of OC_{petro} and the term "$3600 \times 24 \times 365.25$" is the conversion factor from seconds to years.

In 2013 (wet season) and 2019 (wet and dry season), a river sample at AMdD was collected the day after the river sample collected at MLC. Hence, it is possible to compare the instantaneous dissolved Re fluxes measured in between these 2 locations. We can also compare the mean annual dissolved Re fluxes using discharge-weighted average $[\mathrm{Re}]_{\mathrm{diss}}$ in MLC and AMdD. We assume minimal water input between MLC and AMdD and hence consider that $\mathrm{Q}^{\mathrm{MLC}} = \mathrm{Q}^{\mathrm{AMdD}}$. By doing so, we obtain a "minimum" value for the flux of OC_{petro} oxidation in the floodplain. The parameter values and uncertainties are given in the Dataset S5. The uncertainties for "Q" are assumed to be 0 since we consider $\mathrm{Q}^{\mathrm{MLC}} = \mathrm{Q}^{\mathrm{AMdD}}$. Calculation of uncertainty is done by a Monte Carlo simulation (run 10 000 times for each) assuming random distribution of values between the reported positive and negative errors.

The term $\mathrm{J}_{Re-OC}^{\mathrm{meas}}/\mathrm{J}_{Re-OC}^{\mathrm{calc}}$ for this MLC-AMdD section (Fig. 4B) can be calculated as:

$$\frac{\mathrm{J}_{Re-OC}^{\mathrm{meas}}}{\mathrm{J}_{Re-OC}^{\mathrm{calc}}} = \frac{\mathrm{J}_{Re-OC}^{\mathrm{AMdD}}}{\mathrm{J}_{Re-OC}^{\mathrm{MLC}}} \; (Eq.3)$$

The instantaneous $J^{meas}_{Re-OC}/J^{calc}_{Re-OC}$ for this section range from 1.04 to 1.30. These $J^{meas}_{Re-OC}/J^{calc}_{Re-OC}$ values >1 indicates addition of dissolved rhenium from OC_{petro} oxidation in this river section. We interpret this input of dissolved rhenium OC_{petro} derived Re as the result of OW of Andean-derived sediment deposited during the transit of these sediment through the floodplain.

The increase in Re flux can be converted into carbon flux using previously calculated mean annual J^{AMdD}_{Re-OC} and J^{MLC}_{Re-OC}:

$$J^{Floodplains}_{OCpetro-ox}(AMdD - MLC) = \frac{J^{AMdD}_{Re-OC} - J^{MLC}_{Re-OC}}{([Re]/[OC])_{solids}} \quad (Eq. 4)$$

With:

- " $J^{Floodplains}_{OCpetro-ox}(AMdD - MLC)$" the oxidation flux of OC_{petro} (in $MtC.yr^{-1}$) taking place in the floodplain section between MLC and AMdD.
- "$([Re]/[OC])_{solids}$" is the Re/OC ratio in sedimentary rocks being weathered (in $g.g^{-1}$) of 0.93×10^{-7} $g.g^{-1}$ (see section "Re concentrations in solids") in the main text

We calculate a $J^{Floodplains}_{OCpetro-ox}(AMdD - MLC)$ value of 0.03±0.01 $MtC.yr^{-1}$, which is a minimum estimate since we assumed $Q^{MLC} = Q^{AMdD}$.

<u>Section between AMdD and CICRA:</u>
In the section between the confluence with the Rio Manu (AMdD) and CICRA, we can establish a Re mass budget using the approach from previous studies (2–4). We compare the sum of the fluxes for the major tributaries in that section (AMdD, Manu, Chilibe and Colorado) with the measured Re flux at CICRA:

$$\frac{J^{meas}_{Re-OC}}{J^{calc}_{Re-OC}} = \frac{J^{CICRA}_{Re-OC}}{J^{AMdD}_{Re-OC} + J^{Manu}_{Re-OC} + J^{Chilibe}_{Re-OC} + J^{Colorado}_{Re-OC}} \quad (Eq. 5)$$

With J^{CICRA}_{Re-OC} the dissolved Re flux derived from OCpetro oxidation at CICRA, J^{AMdD}_{Re-OC} for the Alto Madre de Dios upstream of the confluence with the Rio Manu, J^{Manu}_{Re-OC} for the Rio Manu, $J^{Chilibe}_{Re-OC}$ for the Rio Chilibe and $J^{Colorado}_{Re-OC}$ for the Rio Colorado.

The Re fluxes are calculated using the same equations as previously (*Eq. 1 and 2*) for wet and dry seasons 2013 (March and August) and 2019 (March and May). The discharge values are derived from Ref. (2) for the 2013 sampling campaigns and from Ref. (4) for the 2019 sampling campaigns.

The mean annual discharge of the Chilibe catchment is unknown. The contribution of the Chilibe to the discharge of the Madre de Dios at CICRA is 16% in March 2013 (2), 6% in August 2013 (2), 3% in March 2019 (4) and 7% in May 2019 (4). Hence the average contribution of the Chilibe to the discharge at CICRA over these 4 sampling campaigns is 7.5%. We use this value to estimate the mean annual discharge of the Chilibe River $Q_{Chilibe} = 162$ m^3/s. All other mean annual discharge values are from Ref. (5) and reported in Table S3.

The instantaneous $J^{meas}_{Re-OC}/J^{calc}_{Re-OC}$ for this section is ~1 for March 2013, March 2019 and May 2019, indicating no gain or loss of Re during floodplain transit. The $J^{meas}_{Re-OC}/J^{calc}_{Re-OC}$ for August 2013 is 0.85 which may indicate loss of dissolved Re during the dry season. We did not attempt to convert the mean annual floodplain Re flux into net carbon flux because the associated uncertainty is too large ($J^{meas}_{Re-OC}/J^{calc}_{Re-OC}$ = 1.07±0.18).

Section between CICRA and Puerto Maldonado (PM):

In the section between CICRA and Puerto Maldonado, we do not have any paired instantaneous Re concentration and discharge, but we can calculate mean annual Re fluxes. In that section, the three main tributaries contributing to the total discharge in Puerto Maldonado are the Madre de Dios at CICRA, the Rio Los Amigos and the Rio Inambari. We compare the sum of the fluxes for the major tributaries in that section (CICRA, Los Amigos, Inambari) with the measured Re flux at Puerto Maldonado:

$$\frac{\mathrm{J}_{Re-OC}^{\mathrm{meas}}}{\mathrm{J}_{Re-OC}^{\mathrm{calc}}} = \frac{\mathrm{J}_{Re-OC}^{\mathrm{PM}}}{\mathrm{J}_{Re-OC}^{\mathrm{CICRA}} + \mathrm{J}_{Re-OC}^{\mathrm{Los\ Amigos}} + \mathrm{J}_{Re-OC}^{\mathrm{Inambari}}} \quad (Eq. 6)$$

For the discharge at Los Amigos, we assume that $Q_{\mathrm{Los\ Amigos}} = Q_{\mathrm{PM}} - Q_{\mathrm{CICRA}} - Q_{\mathrm{Inambari}} = 235\ \mathrm{m^3/s}$

We calculate a mean annual $\mathrm{J}_{Re-OC}^{\mathrm{meas}}/\mathrm{J}_{Re-OC}^{\mathrm{calc}}$ for this section of 0.98±0.15. We did not attempt to convert the mean annual floodplain Re flux into net carbon flux because the associated uncertainty is too large.

Section between Puerto Maldonado (PM) and Riberalta (Ribe):

In the section between Puerto Maldonado, we do not have any paired instantaneous Re concentration and discharge but we can calculate mean annual Re fluxes. In that section, the three main tributaries contributing to the total discharge in Puerto Maldonado are the Madre de Dios at PM, the Rio Las Piedras and the Rio Tambopata. We compare the sum of the fluxes for the major tributaries in that section (PM, Las Piedras, Tambopata) with the measured Re flux at Riberalta:

$$\frac{\mathrm{J}_{Re-OC}^{\mathrm{meas}}}{\mathrm{J}_{Re-OC}^{\mathrm{calc}}} = \frac{\mathrm{J}_{Re-OC}^{\mathrm{Ribe}}}{\mathrm{J}_{Re-OC}^{\mathrm{PM}} + \mathrm{J}_{Re-OC}^{\mathrm{Las\ Piedras}} + \mathrm{J}_{Re-OC}^{\mathrm{Tambopata}}} \quad (Eq. 7)$$

For the discharge of the Madre de Dios at Riberalta (Q_{Ribe}), we use the value from the nearby station El Sena (6).

We calculate a mean annual $\mathrm{J}_{Re-OC}^{\mathrm{meas}}/\mathrm{J}_{Re-OC}^{\mathrm{calc}}$ for this section of 1.11±0.13. We did not attempt to convert the mean annual floodplain Re flux into net carbon flux because the associated uncertainty is too large.

References:

1. K. E. Clark, *et al.*, Erosion of organic carbon from the Andes and its effects on ecosystem carbon dioxide balance. *Journal of Geophysical Research: Biogeosciences* **122**, 449–469 (2017).
2. M. A. Torres, Baronas J. Jotautas, Clark Kathryn E., Feakins Sarah J., West A. Joshua, Mixing as a driver of temporal variations in river hydrochemistry: 1. Insights from conservative tracers in the Andes-Amazon transition. *Water Resources Research* **53**, 3102–3119 (2017).
3. J. J. Baronas, M. A. Torres, K. E. Clark, A. J. West, Mixing as a driver of temporal variations in river hydrochemistry: 2. Major and trace element concentration dynamics in the Andes-Amazon transition. *Water Resources Research* **53**, 3120–3145 (2017).
4. E. I. Burt, *et al.*, Conservative transport of dissolved sulfate across the Rio Madre de Dios floodplain in Peru. *Geology* (2021) https:/doi.org/10.1130/G48997.1 (May 19, 2021).
5. N. Abastos Lara, “Balance hidrico superficial de la cuenca del rio Madre de Dios : Amazonia, Bolivia, Peru” (PHICAB, 1987).
6. P. Vauchel, *et al.*, A reassessment of the suspended sediment load in the Madeira River basin from the Andes of Peru and Bolivia to the Amazon River in Brazil, based on 10 years of data from the HYBAM monitoring programme. *Journal of Hydrology* **553**, 35–48 (2017).

Determination of dissolved Re source contributions: The matlab code used for Monte Carlo calculation of Re sources and uncertainties is paste below.

```
clear all
n = 1e4;
% Load data
Table = readtable('Re-prop.xlsx');
Table = table2array(Table);

% Description of columns content
A = Table(:,1);%[Na]
errA = A*0.05;% Na uncertainties
B = Table(:,2);%[Cl]
errB = B*0.05;% Cl uncertainties
C = Table(:,3);% [SO4]
errC = C*0.05;% SO4 uncertainties
D = Table(:,4);% [Re]
errD = D*0.1;% Re uncertainties

nrows=166;
E=zeros(nrows,1);
F=zeros(nrows,1);
G=zeros(nrows,1);
H=zeros(nrows,1);
I=zeros(nrows,1);
J=zeros(nrows,1);
K=zeros(nrows,1);
errK=zeros(nrows,1);

% Generation of random concentration values within uncertainties
for row = 1:nrows;
A_gen = (A(row,1) + randn(n,1).*(errA(row,1)));
B_gen = (B(row,1) + randn(n,1).*(errB(row,1)));
C_gen = (C(row,1) + randn(n,1).*(errC(row,1)));
D_gen = (D(row,1) + randn(n,1).*(errD(row,1)));

% Calculation of concentrations corrected from precipitation values
E_gen = A_gen - 0.85.*B_gen;% [Na*]
E(row,1) = mean(E_gen);
errE(row,1) = std(E_gen);% Na* uncertainties

F_gen = C_gen - 0.53.*B_gen;% [SO4*]
F(row,1) = mean(F_gen);
errF(row,1) = std(F_gen);% SO4* uncertainties

G_gen = D_gen - 0.0014.*B_gen;% [Re*]
G(row,1) = mean(G_gen);
errG(row,1) = std(G_gen); %Re* uncertainties

% Calculation of [Re]sulfides and uncertainties
H_gen = C_gen.*(2*10^(-4) + (4*10^(-3) - 2*10^(-4)) .* rand(n,1));
H(row,1) = median(H_gen);
errH(row,1) = std(H_gen);

% Calculation of [Re]silicates and uncertainties
```

```
I_gen = A_gen.*(4*10^(-4) + (2*10^(-3) - 4*10^(-4)) .* rand(n,1));
I(row,1) = median(I_gen);
errI(row,1) = std(I_gen);

% Calculation of [Re]OC and uncertainties
J_gen = D_gen - H_gen - I_gen;
J(row,1) = median(J_gen);
errJ(row,1) = std(J_gen);
M(row,1) = mean(J_gen./D_gen);%proportion of Re OC

end
Table(1:nrows,5) = E';
Table(1:nrows,6) = F';
Table(1:nrows,7) = G';
Table(1:nrows,8) = H';
Table(1:nrows,9) = errH';
Table(1:nrows,10) = I';
Table(1:nrows,11) = errI';
Table(1:nrows,12) = J';
Table(1:nrows,13) = errJ';
Table(1:nrows,14) = M';
```

Determination of Re fluxes, OC_{petro} oxidation fluxes and uncertainty: The matlab code used for Monte Carlo calculation of Re fluxes, OC_{petro} oxidation fluxes and uncertainty is paste below.

```
clear all
n = 1e4;

% Load data
Table = readtable('Re_fluxes.xlsx');
Table = table2array(Table);

% Description of columns content
AnnRunoff = Table(:,1); % Annual Runoff
ErrAnnRunoff = Table(:,2);% Uncertainties annual Runoff
Re_Ave = Table(:,3); % Average [Re] (ppt)
ErrRe_Ave = Table(:,4); % StdErr Average [Re]
PropRe_Ave = Table(:,5); % Average fC
ErrPropRe_Ave = Table(:,6); % Uncertainties Average fC
Re_DWAve = Table(:,7); % Discharge weighted Average [Re] (ppt)
ErrRe_DWAve = Table(:,8); % Discharge weighted Average fC
PropRe_DWAve = Table(:,9); % Uncertainties Average fC
Inst_ReFlux = Table(:,10); % Average of instantaneous Re fluxes
ErrInst_ReFlux = Table(:,11); % StdErr

% (Re/OCpetro) Endmembers values
ReOCsolid_min = 0.55*10^-7;
ReOCsolid_max = 1.31*10^-7;

nrows=14;

% Generation of random parameter values within uncertainties
for row = 1:nrows;
AnnRunoff_gen = (AnnRunoff(row,1) + randn(n,1).*(ErrAnnRunoff(row,1)));
```

```
Re_Ave_gen = (Re_Ave(row,1) - 2*ErrRe_Ave(row,1) +
2*rand(n,1).*(2*ErrRe_Ave(row,1)));
Re_DWAve_gen = (Re_DWAve(row,1) - 2*ErrRe_Ave(row,1) +
2*rand(n,1).*(2*ErrRe_Ave(row,1)));
PropRe_Ave_gen = (PropRe_Ave(row,1) - ErrPropRe_Ave(row,1) +
2*rand(n,1).*(ErrPropRe_Ave(row,1)));
PropRe_DWAve_gen = (PropRe_DWAve(row,1) - ErrPropRe_Ave(row,1) +
2*rand(n,1).*(ErrPropRe_Ave(row,1)));
ReOCsolid_gen = (ReOCsolid_min + (ReOCsolid_max - ReOCsolid_min) .*
rand(n,1));
Inst_ReFlux_gen = (Inst_ReFlux(row,1) - 2*ErrInst_ReFlux(row,1)+
2*rand(n,1).*(2*ErrInst_ReFlux(row,1)));

% Calculation of Average Re fluxes and uncertainties
FluxRe_Ave_gen = AnnRunoff_gen .* Re_Ave_gen / 1000 .* PropRe_Ave_gen ./
ReOCsolid_gen / 10^6 ;
FluxRe_Ave(row,:) = quantile(FluxRe_Ave_gen,[.15 .50 .85]);
ErrFluxRe_Ave(row,1) = std(FluxRe_Ave_gen);

% Calculation of Discharge-weigthed Average Re fluxes and uncertainties
FluxRe_DWAve_gen = AnnRunoff_gen .* Re_DWAve_gen / 1000 .*
PropRe_DWAve_gen ./ ReOCsolid_gen / 10^6 ;
FluxRe_DWAve(row,:) = quantile(FluxRe_DWAve_gen,[.15 .50 .85]);
ErrFluxRe_DWAve(row,1) = std(FluxRe_DWAve_gen);

% Calculation of Average instantaneous Re fluxes and uncertainties
FluxRe_InstAve_gen = Inst_ReFlux_gen .* PropRe_Ave_gen ./ ReOCsolid_gen
/ 10^6 ;
FluxRe_InstAve(row,:) = quantile(FluxRe_InstAve_gen,[.15 .50 .85]);
ErrFluxRe_InstAve(row,1) = std(FluxRe_InstAve_gen);

end

FluxRe_Ave
FluxRe_DWAve
FluxRe_InstAve
```

Dataset S1 (Dataset_S1.xls). All measured major ion, rhenium (Re) concentrations and proportions of Re derived from the weathering of each rock source.

Dataset S2 (Dataset_S2.xls). Rhenium and other geochemical concentrations in bedload, bedrock and soil samples.

Dataset S3 (Dataset_S3.xls). Main catchment characteristics, discharge, runoff, Re yields and rates of OC_{petro} oxidation (JOCpetro-ox) calculated using different methods

Dataset S4 (Dataset_S4.xls). Rock weathering end-members compositions

Dataset S5 (Dataset_S5.xls). Mass-balance calculation of instantaneous (in March and August 2013 and in March and May 2019) and mean annual Re yield of the main Madre de Dios tributaries
